# PCEtoFHIR: Decomposition of Postcoordinated SNOMED CT Expressions for Storage as HL7 FHIR Resources

**DOI:** 10.2196/57853

**Published:** 2024-09-17

**Authors:** Tessa Ohlsen, Josef Ingenerf, Andrea Essenwanger, Cora Drenkhahn

**Affiliations:** 1 IT Center for Clinical Research University of Luebeck Luebeck Germany; 2 Institute of Medical Informatics University of Luebeck Luebeck Germany; 3 mio42 GmbH Berlin Germany

**Keywords:** SNOMED CT, HL7 FHIR, TermInfo, postcoordination, semantic interoperability, terminology, OWL, semantic similarity

## Abstract

**Background:**

To ensure interoperability, both structural and semantic standards must be followed. For exchanging medical data between information systems, the structural standard FHIR (Fast Healthcare Interoperability Resources) has recently gained popularity. Regarding semantic interoperability, the reference terminology SNOMED Clinical Terms (SNOMED CT), as a semantic standard, allows for postcoordination, offering advantages over many other vocabularies. These postcoordinated expressions (PCEs) make SNOMED CT an expressive and flexible interlingua, allowing for precise coding of medical facts. However, this comes at the cost of increased complexity, as well as challenges in storage and processing. Additionally, the boundary between semantic (terminology) and structural (information model) standards becomes blurred, leading to what is known as the TermInfo problem. Although often viewed critically, the TermInfo overlap can also be explored for its potential benefits, such as enabling flexible transformation of parts of PCEs.

**Objective:**

In this paper, an alternative solution for storing PCEs is presented, which involves combining them with the FHIR data model. Ultimately, all components of a PCE should be expressible solely through precoordinated concepts that are linked to the appropriate elements of the information model.

**Methods:**

The approach involves storing PCEs decomposed into their components in alignment with FHIR resources. By utilizing the Web Ontology Language (OWL) to generate an OWL ClassExpression, and combining it with an external reasoner and semantic similarity measures, a precoordinated SNOMED CT concept that most accurately describes the PCE is identified as a Superconcept. In addition, the nonmatching attribute relationships between the Superconcept and the PCE are identified as the “Delta.” Once SNOMED CT attributes are manually mapped to FHIR elements, FHIRPath expressions can be defined for both the Superconcept and the Delta, allowing the identified precoordinated codes to be stored within FHIR resources.

**Results:**

A web application called PCEtoFHIR was developed to implement this approach. In a validation process with 600 randomly selected precoordinated concepts, the formal correctness of the generated OWL ClassExpressions was verified. Additionally, 33 PCEs were used for two separate validation tests. Based on these validations, it was demonstrated that a previously proposed semantic similarity calculation is suitable for determining the Superconcept. Additionally, the 33 PCEs were used to confirm the correct functioning of the entire approach. Furthermore, the FHIR StructureMaps were reviewed and deemed meaningful by FHIR experts.

**Conclusions:**

PCEtoFHIR offers services to decompose PCEs for storage within FHIR resources. When creating structure mappings for specific subdomains of SNOMED CT concepts (eg, allergies) to desired FHIR profiles, the use of SNOMED CT Expression Templates has proven highly effective. Domain experts can create templates with appropriate mappings, which can then be easily reused in a constrained manner by end users.

## Introduction

### Background

The growing digitization of medical records has led to an increase in patient data available for health care analysis. These data must be utilized to improve medical care and offer more personalized treatments. However, to accomplish this, the ability to automatically exchange and process data between different systems is essential. This requires not only technical compatibility but also semantic interoperability, ensuring that the data’s meaning is preserved when transferred to another system. The ability to exchange and utilize data across different systems is crucial for fully leveraging digital medical records and improving patient care [1].

To ensure semantic interoperability, it is essential to follow both structural and semantic standards. Structural standards specify the syntax for accessing data fields within information models. In recent years, the newly developed HL7 (Health Level 7) standard, FHIR (Fast Healthcare Interoperability Resources), has gained international recognition due to its emphasis on simplified implementation and the use of modern technologies [2]. Semantic standards, by contrast, involve terminologies that use language-independent codes to represent the meaning of data in an interoperable manner. SNOMED CT is widely recognized as the most comprehensive medical terminology for enhancing semantic interoperability [3]. In 2021, Germany acquired a national license for SNOMED CT, leading to increased interest and usage of the terminology. SNOMED CT concepts are now being integrated into data modeling efforts, such as the Medical Information Objects by the German National Association of Statutory Health Insurance Physicians (NASHIP) [4] and the core data set of the Medical Informatics Initiative (MII) [5]. The use of SNOMED CT, which contains over 350,000 concepts, aims to provide a machine-readable interlingua that minimizes coding issues specific to different countries and medical fields. However, due to the complexity of natural language, not all medical situations can be accurately coded using SNOMED CT’s extensive set of precoordinated concepts. To address this and avoid a rapid increase in the number of new concepts, SNOMED CT, unlike many other vocabularies, supports postcoordination. This feature allows the combination of precoordinated concepts into new expressions using a formal grammar.

Therefore, postcoordination is a unique feature that significantly enhances the precision of medical documentation and greatly increases SNOMED CT’s expressive power. However, the adoption of postcoordination has been slow due to various challenges. While some of these obstacles have already been addressed [6-9], integrating postcoordinated expressions (PCEs) into the electronic health records of legacy hospital information systems remains difficult for several reasons (Textbox 1).

**Obstacles to integrating postcoordinated SNOMED Clinical Terms expressions into electronic health records.**

- **Adherence to familiar data structures**

Medical circumstances are usually documented using individual codes, and there are established methods for storing and processing these codes. While restrictions such as length-limited data types can be managed with simple codes, they pose challenges when dealing with arbitrarily large formal expressions, such as postcoordinated expressions (PCEs). This has led to concerns about the practicality of using PCEs [10].

- **Lack of technical support**

The technical handling of PCEs is inherently complex, requiring at a minimum a description logic reasoner and the implementation of several formal specifications, such as the Concept Model, Compositional Grammar, and Expression Constraint Language, as defined by SNOMED International [3]. While specialized terminology servers can help alleviate the implementation burden, currently only the CSIRO (Commonwealth Scientific and Industrial Research Organization) Ontoserver [11] supports postcoordination [12].

- **Difficulties with FHIR (Fast Healthcare Interoperability Resources) search**

When searching for information in FHIR resources, postcoordination is supported only if the exact same PCE has been explicitly and previously defined in a FHIR CodeSystem supplement. These supplements enable the extension of the standard FHIR CodeSystem for SNOMED CT with a collection of PCEs (eg, for value set definitions), but they do not support the recording of PCEs in a patient’s electronic health record.

Given that these challenges are unlikely to be resolved in the short term, a different approach is needed to ensure interoperability between systems that support postcoordination and those that do not. Consequently, this paper will present an alternative representation of PCEs.

**Figure 1:**
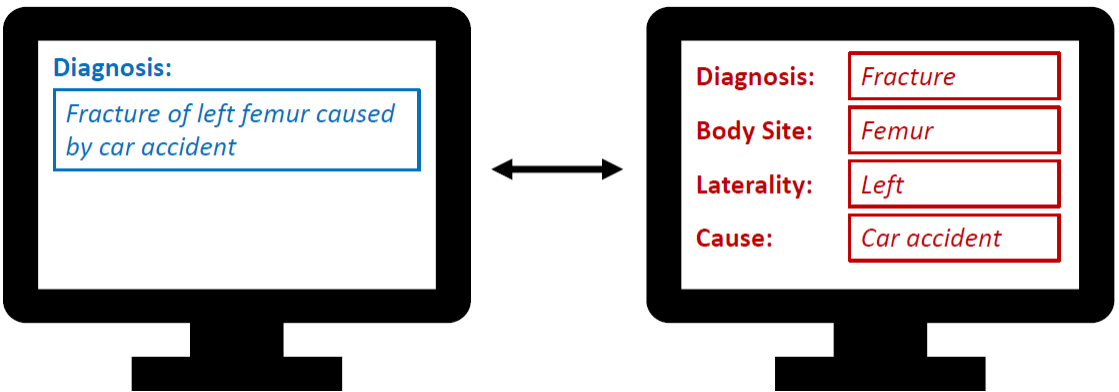
TermInfo: The same medical fact can be represented using either the terminology (left side) or the information model (right side) more heavily.

It has long been recognized that there is significant overlap between the scopes of structural and semantic standards, leading to unclear responsibilities and potentially ambiguous representations of medical facts, as well as inconsistent redundancies. This issue, known as the TermInfo problem [3,13-15], largely arose from the independent development of these standards, which resulted in mutual coverage of the same data elements (Figure 1). SNOMED CT’s postcoordination capability further blurs the distinction between structure and semantics, potentially exacerbating the existing problem. However, PCEs are fully interpretable, allowing the information components they contain to be identified and flexibly disassembled. Building on extensive knowledge from previous projects [6,16-18] and the current integration of SNOMED CT with FHIR, the authors propose PCEtoFHIR—an application designed to decompose PCEs for storage as FHIR resources in a manner that preserves their meaning.

### Related Work

Although there is a growing body of literature on the postcoordination of SNOMED CT concepts [6,16,19-21], a literature search revealed no existing publications specifically addressing the storage of PCEs in FHIR resources. However, a Health Level Seven International (HL7) working group is addressing this topic and provides several resources. For selected FHIR resources, such as Condition and Observation, mappings of some SNOMED CT attributes to FHIR are currently available (see [22] and [23]). Additionally, the Confluence pages “SNOMED on FHIR” (“Bindings to FHIR Clinical Resources”) [24] outline various options for mapping SNOMED CT attributes to FHIR while avoiding semantic overlaps. The information from these documents is considered in our work.

Additionally, some papers discuss the use of SNOMED CT in combination with standardized information models based on the HL7 Reference Information Model (RIM), HL7 Clinical Document Architecture, and HL7 FHIR resources. For instance, a project by Perez-Rey et al [25] focused on linking the normal form of precoordinated SNOMED CT concept definitions, normalizing SNOMED CT concepts, and binding them to HL7 RIM classes. This approach could potentially be extended to PCEs. However, the HL7 version 3 standard has not been widely adopted due to its complexity [1]. A project by Arguello-Casteleiro et al [26] addressed mapping precoordinated SNOMED CT concepts or PCEs from Consolidated Clinical Document Architecture to FHIR resources. While the objectives of this approach are similar to those of our work, Arguello-Casteleiro et al focused heavily on ontology. By contrast, our work primarily utilizes the widely adopted FHIR and SNOMED CT native specifications. Additionally, the specific ocular diseases examined by Arguello-Casteleiro et al pertain to a very narrow subset of SNOMED CT expressions. Further, the publication by Arguello-Casteleiro et al does not provide information on the extent to which all the data included in the PCEs can be transferred into the FHIR representation.

## Methods

### Overview

**Figure 2:**
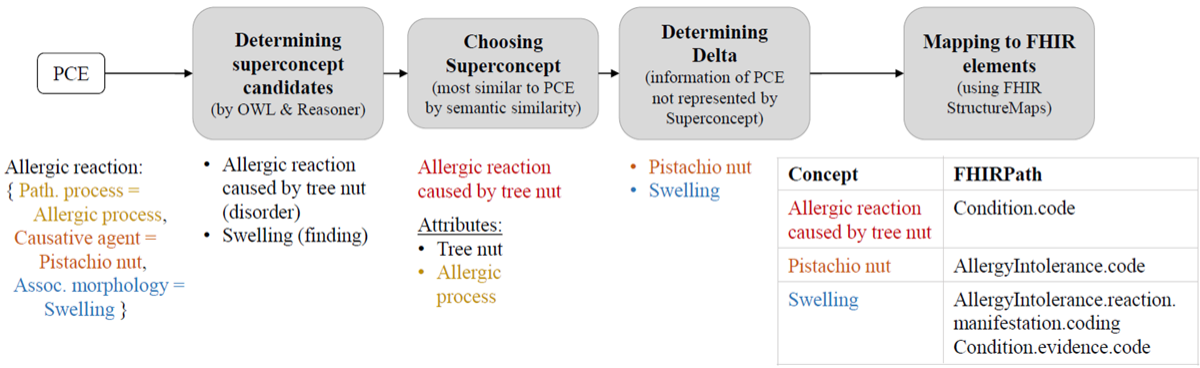
The decomposition of a PCE into elements of (profiled) FHIR resources consists of 4 steps. FHIR: Fast Healthcare Interoperability Resources; OWL: Web Ontology Language; PCE: postcoordinated expression.

This work aims to develop and implement an approach that enables the storage of a SNOMED CT PCE within FHIR resources using only precoordinated codes. In this alternative representation, the PCE will be decomposed into precoordinated concepts, which can then be stored in appropriate elements of corresponding FHIR resources. An overview of the proposed approach is shown in Figure 2.

A PCE, once verified for syntactic and semantic correctness, serves as the input. This PCE can be classified within SNOMED CT using the Web Ontology Language (OWL) and a reasoner, allowing for the identification of its direct supertype ancestors. Among these concepts, the most similar one to the PCE is selected as the Superconcept. The Delta is then calculated between the Superconcept and the PCE, encompassing all the information in the PCE that is not represented by the Superconcept. In the final step, suitable elements of corresponding FHIR resources must be identified to store the information of the Superconcept and the Delta. To facilitate this, FHIR StructureMaps that define these associations on a general level need to be created in advance.

### Validation of PCE

To ensure that flexible PCEs can be accurately interpreted and evaluated, they must adhere to the syntactic requirements of the Compositional Grammar and the semantic rules of the Concept Model defined by SNOMED International. In the initial step, the input PCE is checked for syntactic and semantic correctness using the HL7 FHIR service *$validate-code* and Ontoserver. Ontoserver, a FHIR-based terminology server provided by the Australian Commonwealth Scientific and Industrial Research Organization (CSIRO), supports and facilitates working with key coding systems such as SNOMED CT and LOINC (Logical Observation Identifiers Names and Codes) [11]. Additionally, Ontoserver provides an integrated description logic reasoner and full support for SNOMED CT postcoordination, enabling PCE validation directly on the Ontoserver [11]. Only if a PCE is both syntactically and semantically correct will the process automatically proceed to the next steps.

### Determining Superconcept Candidates

OWL serves as an exchange format for ontologies and is used here to determine the ancestors of a PCE. OWL allows for the representation of complex knowledge about concepts and their interrelationships [27,28]. SNOMED CT can also be represented as an OWL ontology, with the definitions of individual SNOMED CT concepts provided as OWL expressions in the monthly release packages of SNOMED CT, alongside other formats [29].

To obtain SNOMED CT as an OWL ontology, the SNOMED OWL Toolkit [30] was used in the Release Format 2 of the International Edition, dated 2023-04-30 [31]. The generated ontology includes, among other components, precoordinated SNOMED CT concepts and their definitions in functional syntax.

**Figure 3:**
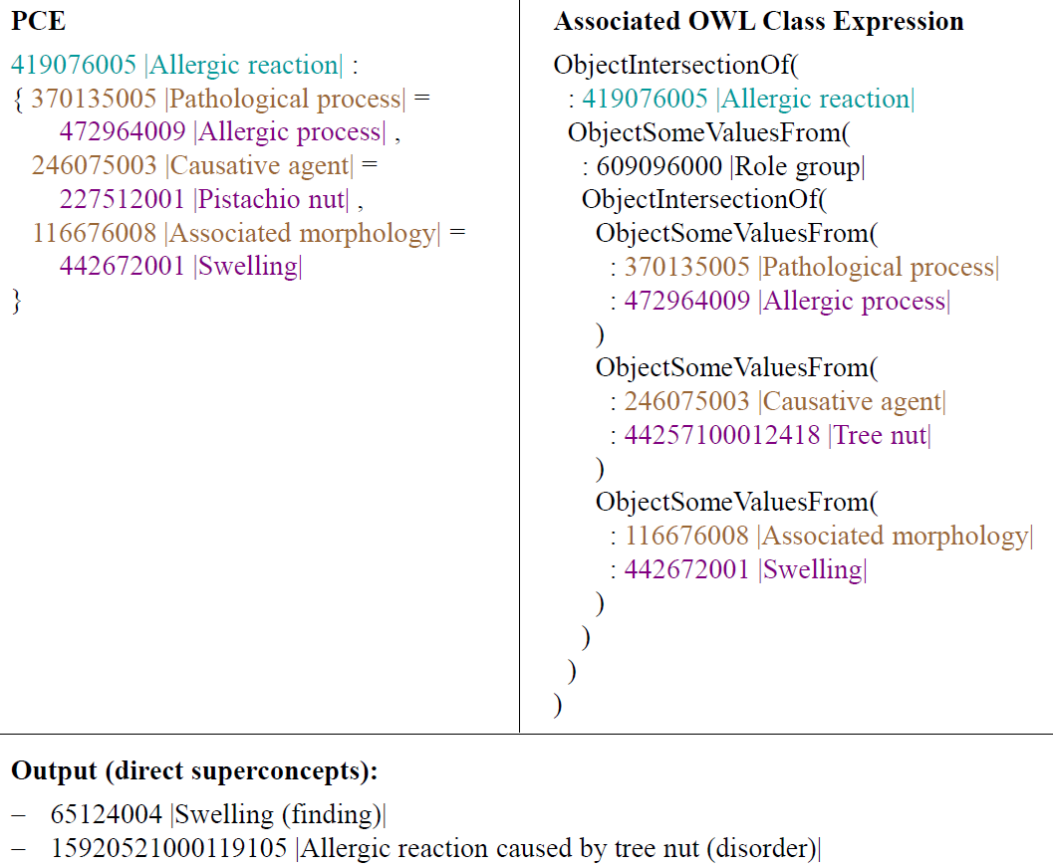
PCE on the left side and the associated OWL ClassExpression based on functional syntax on the right side. OWL: Web Ontology Language; PCE: postcoordinated expression.

PCEs, by contrast, are based on the syntax of the Compositional Grammar. Therefore, a PCE must be transformed into an OWL ClassExpression for further processing. For each component of the PCE, the corresponding OWL counterpart is determined based on the existing concept definitions, as shown in Table 1. An algorithm utilizing the Java (Oracle Corporation) library OWL API [32] is developed to automate this transformation. The result of the algorithm is an OWL ClassExpression structured similarly to the OWL ClassExpressions created by SNOMED International for SNOMED CT concept definitions. An exemplary OWL expression for a PCE is shown in Figure 3, with Fully Specified Names added for improved readability. The symbol “:” serves as a placeholder for the defined namespace, which in this case is “http://snomed.info/id/” [29].

**Table 1:** Various OWL^a^ constructs are used for the representation of the components of a PCE^b^. While the components in the first 2 rows are exclusively used in the native OWL ontology of SNOMED CT^c^, the OWL constructs below are also used for the transformation of PCEs.

PCE component	OWL construct
SNOMED CT concept	OWL Class
SNOMED CT attribute, attribute value: SNOMED CT concept	OWL ObjectProperty
Linking of ungrouped attribute relationships	OWL ObjectIntersectionOf
Linking between individual attribute relationships in a Role Group	OWL ObjectIntersectionOf
Linking between focus concept and all attribute relationships of ungrouped attributes and Role Groups	OWL ObjectIntersectionOf
Attribute relationship of attribute and SNOMED CT Identifier as attribute value	OWL ObjectSomeValuesFrom
All grouped attribute relationships and Role Group Identifier	OWL ObjectSomeValuesFrom

^a^OWL: Web Ontology Language.

^b^PCE: postcoordinated expression.

^c^SNOMED CT: SNOMED Clinical Terms.

In the next step, the previously formed OWL ontology of SNOMED CT, the created PCE-specific OWL ClassExpression, and a description logic reasoner are used to classify the PCE within the existing SNOMED CT hierarchy. A reasoner generates new knowledge through logical inferences from the existing content of an ontology, such as determining superrelationships between concepts [27]. In this work, the ELK reasoner is used because it is one of the few reasoners capable of handling the extensive SNOMED CT ontology [33].

The direct ancestors of PCEs are determined using the ELK reasoner with the Java library elk-reasoner [33]. This process identifies at least one OWL Class corresponding to a precoordinated SNOMED CT concept. All identified precoordinated SNOMED CT concepts are considered potential Superconcept candidates.

### Choosing Superconcept

#### Overview

One of the previously identified Superconcept candidates must be selected as the Superconcept. The Superconcept is the precoordinated SNOMED CT concept that most closely resembles the PCE, covering the largest portion of the information contained within the PCE compared with any other precoordinated SNOMED CT concept.

#### Semantic Similarity Measure

To identify the most similar concept, the semantic similarity between concepts is assessed. This measure calculates the taxonomic proximity between two elements within a knowledge base, such as SNOMED CT. Higher semantic similarity indicates that the two elements are more closely related [34].

While several semantic similarity measures have been proposed in the literature, this work uses a path-based approach developed by Sánchez and Batet [34]. This method is specifically designed for large knowledge bases with a subtype-relationship–based polyhierarchy, such as SNOMED CT [34]. The equation for the calculation is as follows:



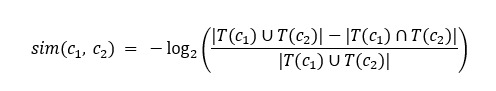



where *T*(*c_i_*) is the set of all ancestors of a concept *c_i_* and the concept *c_i_* itself. To calculate the semantic similarity between two concepts *c_1_* and *c_2_*, the ratio between the set of nonshared ancestors (nominator) and the union of all ancestors of both concepts (denominator) is considered [34].

In our work, the semantic similarity between the classified PCE and each of its Superconcept candidates needs to be calculated. Therefore, the equation is modified as follows:




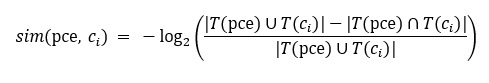




with *c_i_* being one of the Superconcepts. The calculated similarities for the exemplary PCE are shown in Table 2.

**Table 2:** To choose the most fitting Superconcept, the semantic similarity between the PCE^a^ and each of its Superconcept candidates is calculated using the measure proposed by Sánchez and Batet [34]. The previously introduced exemplary PCE shares a larger ratio of ancestors with *Allergic reaction caused by tree nut* than with *Swelling*. Thus, the former leads to a higher semantic similarity and is determined as Superconcept.

Superconcept candidates	Nonshared ancestors	Union of all ancestors	Semantic similarity
Allergic reaction caused by tree nut	8	30	2.10
Swelling	29	30	0.10

^a^PCE: postcoordinated expression.

#### Implementation

An algorithm was developed to select the most suitable (ie, the most semantically similar) SNOMED CT concept from the Superconcept candidates. The calculation of semantic similarity is based on the graph-based approach by Sánchez and Batet [34], as described above. To achieve this, SNOMED CT must first be transformed into a directed acyclic graph (DAG). The DAG was constructed by algorithmically processing the Release Format 2, version 2023-04-30 [31], of SNOMED CT using the Python (Python Foundation) library NetworkX [35]. In the resulting graph, SNOMED CT concepts are represented as nodes, while their relations, including relation types, form the connecting edges.

**Figure 4:**
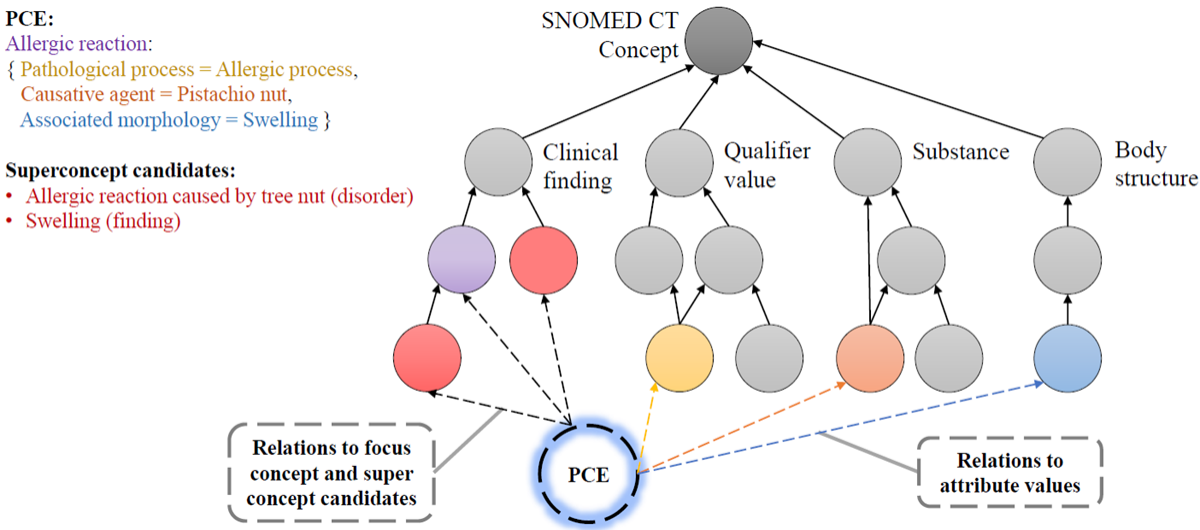
The PCE is represented as a subconcept of its focus concept and the Superconcept candidates. It also has edges to its attribute values. PCE: postcoordinated expression; SNOMED CT: SNOMED Clinical Terms.

To enable semantic similarity calculation, the PCE must be temporarily inserted into the DAG. For this purpose, a node called “pce” is introduced into the graph as a subnode of its focus concept and the previously determined Superconcept candidates (Figure 4). The algorithm iterates over the individual attribute values of the PCE and inserts edges between these attributes and the “pce” node.

Next, the semantic similarity between the PCE and all potential Superconcept candidates is calculated based on the DAG. The Superconcept candidate with the highest semantic similarity describes the PCE most accurately among all precoordinated SNOMED CT concepts. This concept is then defined as the Superconcept.

### Determining Delta

**Figure 5:**
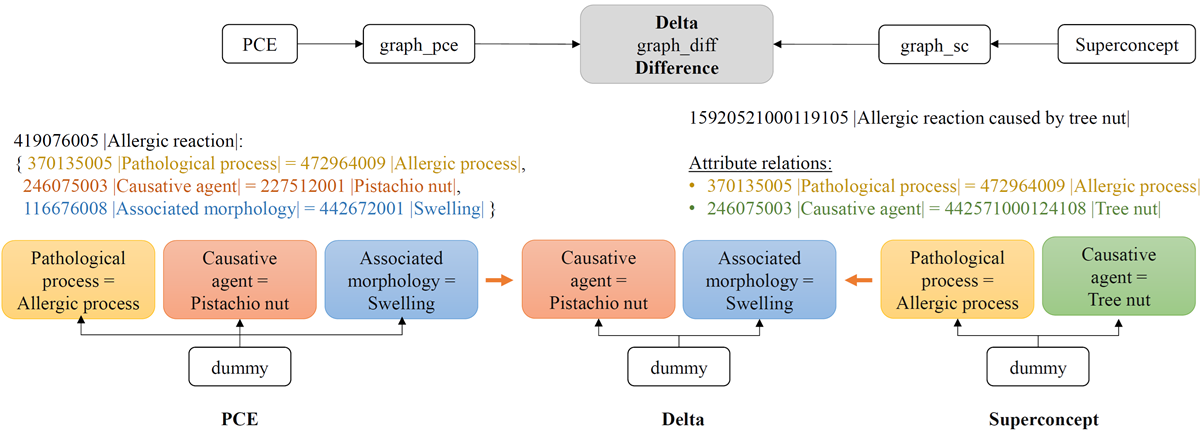
When calculating the delta, the graph of the Superconcept is subtracted from that of the PCE. The determined edges contain attribute relations that were not covered by the Superconcept but are necessary for the precise representation of the PCE. PCE: postcoordinated expression.

Although the Superconcept is the most similar precoordinated concept available in SNOMED CT, it does not cover all the information contained within the PCE. Therefore, the missing parts of information, referred to as the “Delta” between the PCE and the Superconcept, need to be determined. This is achieved using a graph-based approach, where both the attribute relations of the PCE and those of the Superconcept are represented in separate graphs. To facilitate merging later, a dummy root node with the same name is introduced into both graphs. Next, the attribute relations of the PCE and the Superconcept are added as nodes and connected via edges, as shown on either side of Figure 5. The Delta is then calculated by subtracting the edges of the Superconcept graph from the PCE graph. This process eliminates all equivalent components present in both graphs, leaving only the attribute relationships that are not or not precisely represented in the Superconcept graph (see the Delta graph in the middle of Figure 5).

### Mapping to FHIR Elements

In the final step, the Superconcept and the Delta must be stored as precoordinated concepts in suitable FHIR elements. This can be accomplished using either the base FHIR resources from HL7 or more specific FHIR profiles. FHIR profiles are customized versions of FHIR resources tailored to national characteristics, legislation, or specific use cases [35].

In our work, 2 sets of FHIR profiles were considered as target representations for the mapping:

- profiles of the NASHIP (version 1.4.0, based on FHIR R4) [4] and
- profiles of the core data set of the German MII (version 1.0, based on FHIR R4) [5].

To accurately map a decomposed PCE to these profiled FHIR resources, mapping rules are required to align its Superconcept and Delta with the appropriate FHIR elements. As PCEs are often highly individualized, they can be categorized according to their focus concept, attribute relationships, and SNOMED CT Expression Templates. Consequently, it is not necessary to define a mapping rule for each PCE; instead, PCEs with similar content can be mapped using a uniform set of rules.

To demonstrate the applicability of our approach, we consider 5 content categories of PCEs from the top-level hierarchies *Procedure* and *Clinical finding*. These categories include *General procedures* for *Procedure* and *Allergies, Diseases due to allergy, Allergic reactions,* and *General clinical findings* for *Clinical finding*. To ensure the unambiguous assignment of PCEs, SNOMED CT’s Expression Constraint Language (ECL) is used to formally define the scope of each category, creating pairwise disjoint partitions of the two hierarchies.

For each combination of 1 of the 5 content categories and either of the 2 sets of target representations, mapping rules are manually defined based on the respective Expression Templates [36], concept definitions of related precoordinated concepts, existing documents (as described in the “Related Work” section), and the author’s expertise. These rules are formally transcribed into FHIR StructureMaps (R4) [37] using the Java library HAPI FHIR [38]. The resulting 10 FHIR StructureMaps are stored on a local HAPI FHIR server [39].

**Figure 6:**
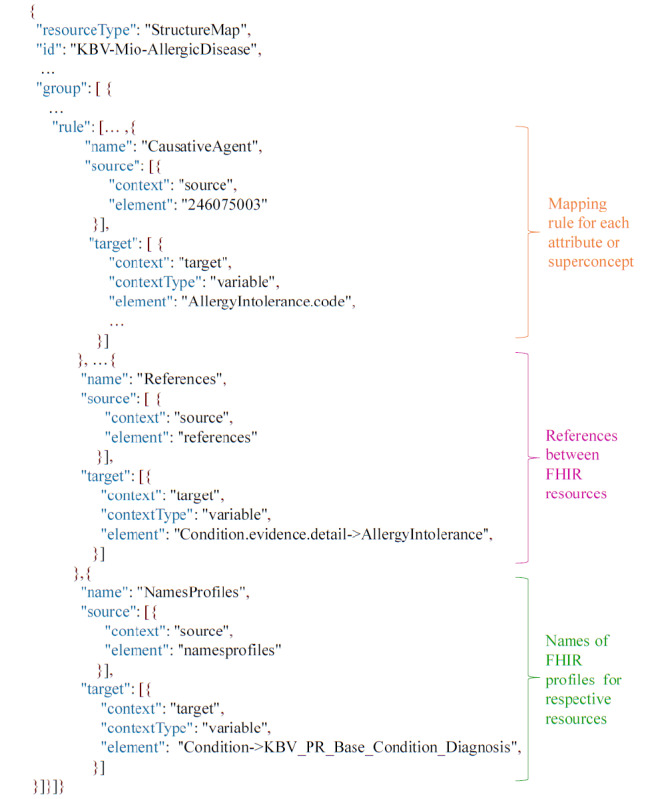
Extract of the FHIR StructureMap for allergic reactions in JSON format. It contains an entry for each SNOMED CT attribute and the Superconcept as well as references and names of the profiles. FHIR: Fast Healthcare Interoperability Resources; SNOMED CT: SNOMED Clinical Terms.

An excerpt of the FHIR StructureMap for the category “Allergic reaction” and the NASHIP profiles is shown in Figure 6. Each StructureMap contains exactly a single “rule” entry for the Superconcept and each relevant attribute relationship for the category. The mapping rule’s “source” is either the string “Superconcept” or the SNOMED CT identifier of the respective attribute. For the “target” element, FHIRPath is used to traverse the FHIR resources and explicitly identify the correct subelement [2]. Illustrated in orange in Figure 6, the mapping of the attribute “Causative agent” (“246075003”) to the FHIRPath *AllergyIntolerance.code* is exemplified. This specifies that the attribute value associated with the PCE should be stored in the code element of the *AllergyIntolerance* resource.

Apart from the central mapping rules, some further information is included in the FHIR StructureMaps.

If necessary, references between FHIR elements and resources are indicated (Figure 6, in pink). Additionally, the FHIR resources associated with the FHIRPath entries are mapped to the names of the respective profiles (Figure 6, in green).

With these general preparations completed, individual decomposed PCEs can now be mapped to appropriate FHIR resources. First, the PCE is assigned to 1 of the 5 content categories by identifying the subsuming subset using the ECL definitions and Ontoserver. Based on the category and desired target representation, the correct FHIR StructureMap is selected. The mapping rules in the StructureMap are then automatically applied to the PCE’s Superconcept and Delta, determining the combinations of FHIR elements and precoordinated SNOMED CT concepts needed for the alternative representation.

### Ethics Approval

This research neither involves human nor animal subjects so ethics approval was not required.

## Results

### Web Application

**Figure 7:**
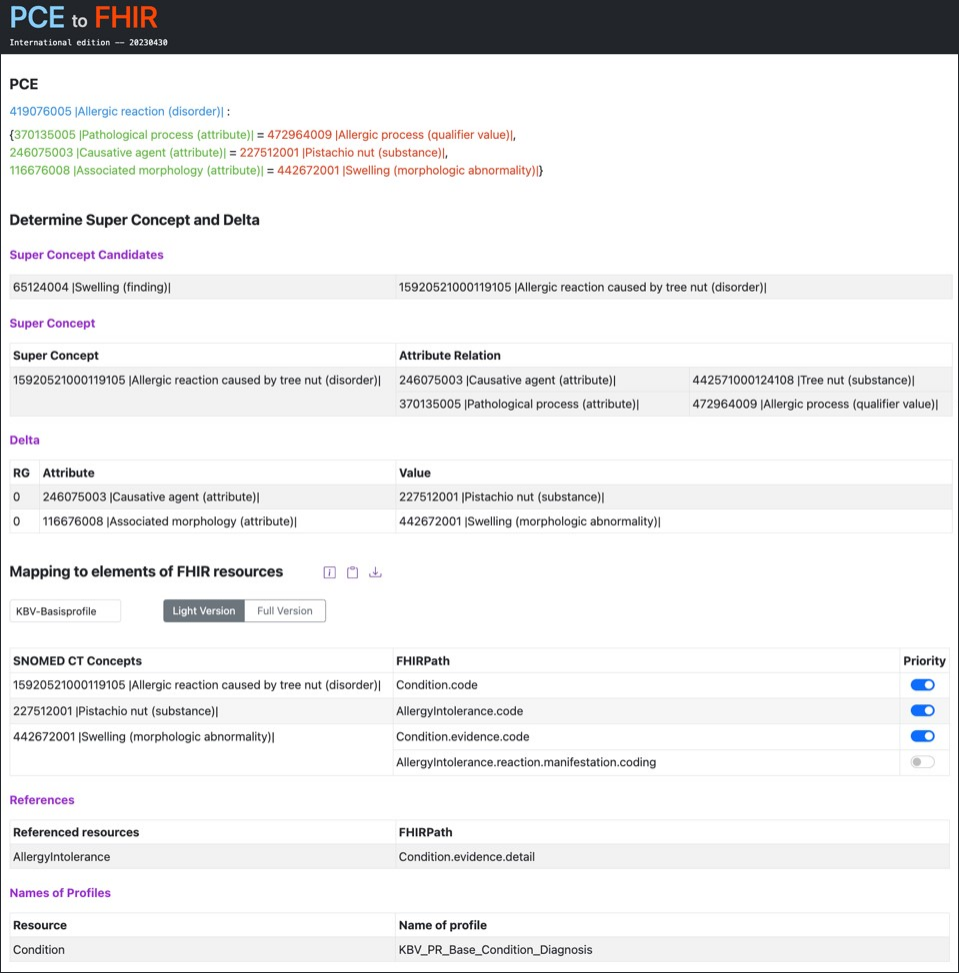
Excerpt of the web application PCEtoFHIR. FHIR: Fast Healthcare Interoperability Resources; PCE: postcoordinated expression.

Bringing all the previously explained preliminary considerations and processing steps together, a web application called “PCEtoFHIR” was developed (Figure 7). This single-page web application was built using Angular (version 15.2.2; Google LLC/Alphabet Inc.) and the Java framework Spring Boot (version 2.7.2; Java version 17).

An excerpt of the web application is shown in Figure 7. After the entered PCE has been checked for syntactic and semantic correctness, the Superconcept candidates, the Superconcept, and the Delta are determined automatically. This information is displayed to the user in the “Determine Super Concept and Delta” section. The Superconcept and Delta are then mapped to FHIR elements according to the category-specific FHIR StructureMaps. The appropriate StructureMap is automatically determined for the PCE based on its content category and the desired mapping target is selected in a combo box. Figure 6 illustrates the target mapping category “KBV-Basisprofile” (KBV Base Profiles; Kassenärztliche Bundesvereinigung [KBV] is the German abbreviation for the NASHIP). Using the StructureMap’s mapping rules, the FHIRPaths, corresponding SNOMED CT concepts, required references, and profile names are displayed and can be copied to the clipboard or downloaded as a text file. This information is shown in Figure 7 in the section “Mapping to elements of FHIR resources.”

The source code of PCEtoFHIR, the created FHIR StructureMaps, and all validation results as described in the following paragraphs are available on GitHub [40].

### Determining Superconcept Candidates

To ensure the correct functionality of the algorithm for generating OWL ClassExpressions, validation was performed with 600 randomly selected precoordinated SNOMED CT concept definitions. These definitions, from the monthly release 2023-04-30 [31], were initially examined for diverse structures and then transformed into OWL ClassExpressions using PCEtoFHIR’s regular algorithm. The generated OWL ClassExpressions was compared with the official OWL ClassExpressions of the concepts, available as part of the same SNOMED CT release, as reference data.

The comparison involved checking whether the OWL ClassExpressions matched syntactically and semantically. Focus concepts were excluded from the semantic validation because the reference data’s OWL ClassExpressions are based on Stated Concept Definitions, while our approach uses Inferred Concept Definitions. Thus, the validation focused solely on whether the focus concepts were syntactically in the correct position within the OWL ClassExpression.

In summary, the comparison revealed no discrepancies between the OWL ClassExpressions generated by PCEtoFHIR and those from the reference data.

### Choosing Superconcept

As described previously, a path-based measure by Sánchez and Batet [34] is applied to calculate semantic similarity in our work. A preliminary analysis was conducted to ensure both the theoretical and practical applicability of this measure.

SNOMED CT’s most striking characteristics include its reliance on subtype relationships and the resulting, heavily interwoven polyhierarchy. Using a path-based approach that incorporates each concept’s ancestors, these central features are prioritized. While several path-based semantic similarity measures are available [34], they mostly rely on the shortest path between concepts [41-44]. Sánchez and Batet [34] argue that in large knowledge bases such as SNOMED CT, a concept inherits information from several hierarchies and is connected to many concepts simultaneously. Therefore, considering only the shortest path is insufficient.

In their proposed measure, Sánchez and Batet [34] consider the ratio between the set of nonshared ancestors and the set of all ancestors of both concepts. Adapted to our approach, this means that for the PCE and each of its Superconcept candidates, all ancestors in the SNOMED CT hierarchy are determined, and the ratio is calculated. Thus, this measure is applicable in principle.

To evaluate if the calculated values are reasonable beyond that, a practical validation by means of an exemplary sample was done in succession. For 33 PCEs taken from a publication by Kate [45] (see detailed explanation in the “Overall Evaluation With Existing PCEs” section), the most similar Superconcept candidate was determined manually and compared with the Superconcept calculated by PCEtoFHIR via Sánchez and Batet’s semantic similarity measure. In 76% (25/33) of the cases, the same Superconcept was chosen [34]. An analysis of the remaining 24% (8/33) revealed that different information components within the PCE were prioritized during Superconcept selection (eg, favoring localization over procedure type), but the divergent choices made by the algorithm were considered plausible. Consequently, the measure by Sánchez and Batet [34] was found to be feasible for Superconcept determination and is therefore used to calculate semantic similarity in this work.

### Mapping to FHIR Elements

As previously described, the Basisprofile of the NASHIP (version 1.4.0) [4] and the profiles of the Core Data Set of the MII (version 1.0) [5] were used for mapping to the FHIR profiles. To illustrate the mapping, 5 categories were considered. For each category and profile type, a FHIR StructureMap was created, resulting in a total of 10 FHIR StructureMaps. Table 3 shows the FHIR resources used for the mapping.

**Table 3:** Utilization of different FHIR^a^ resources per profile type, organized by content category.

Category and profile type		FHIR resources
**Allergies**		
	NASHIP^b^	AllergyIntolerance
	MII^c^	Condition, Observation
**Disease due to allergies**		
	NASHIP	AllergyIntolerance, Condition
	MII	Condition, Observation
**Allergic reaction**		
	NASHIP	AllergyIntolerance, Condition
	MII	Condition, Observation
**Clinical finding (general)**		
	NASHIP	AllergyIntolerance, Condition
	MII	Condition, Observation
**Procedure**		
	NASHIP	Procedure
	MII	Procedure

^a^FHIR: Fast Healthcare Interoperability Resources.

^b^NASHIP: National Association of Statutory Health Insurance Physicians.

^c^MII: Medical Informatics Initiative.

For the category of general *Clinical finding*, the FHIR StructureMaps include mappings for the Superconcept and the following 5 SNOMED CT attributes: *Causative agent, Finding site, Associated morphology, Pathological process,* and *Clinical course.* In addition to the 5 SNOMED CT attributes, the following attributes were considered for the remaining *Clinical finding* categories focusing on allergies: *Has realization, Occurrence,* and *Due to.* Furthermore, for the hierarchy *Procedure*, mapping rules for the Superconcept and the following 12 additional SNOMED CT attributes are established: *Method, Procedure site—Direct, Procedure site—Indirect, Direct substance, Direct morphology, Using substance, Using device, Using access device, Has intent, Access, Surgical approach,* and *Has focus.*

Table 4 shows an example of the mapping of SNOMED CT elements to FHIRPath for Allergic reactions. The complete set of mapping rules and the associated StructureMaps are available online [46].

**Table 4:** The SNOMED CT elements and the associated FHIRPath for the category “Allergic reaction” based on the profiles of the NASHIP^a^.

SNOMED CT element	FHIRPath in NASHIP profiles
Super concept	Condition.code
Causative agent	AllergyIntolerance.code
Finding site	Condition.bodySite
Associated morphology	AllergyIntolerance.reaction.manifestation.coding:snomed Condition.evidence.code
Pathological process	AllergyIntolerance.reaction.manifestation.coding:snomed Condition.evidence.code
Has realization	AllergyIntolerance.reaction.manifestation.coding:snomed Condition.evidence.code
Occurrence	AllergyIntolerance.extension: abatement-lebensphase-von [47]
Clinical course	Extension of HL7: Condition.condition-diseaseCourse [48]
Due to	Extension of HL7: Condition.condition-dueTo [49]

^a^NASHIP: National Association of Statutory Health Insurance Physicians.

To ensure their correctness, the created FHIR StructureMaps were validated by author AE, who had not been involved in the PCEtoFHIR project up to that point. AE possesses in-depth knowledge of FHIR and SNOMED CT and is profoundly familiar with the MII and NASHIP profiles due to her former and current work. She validated the FHIR StructureMaps based on the definitions of the profiles used, ensuring the correct choice of profiles, the accurate mapping of SNOMED CT elements to FHIRPaths, and appropriate references. The validation yielded the following results: the choice of profiles and references was found to be entirely correct. The mapping rules between SNOMED CT elements and FHIR paths were largely correct as well; however, 8 suggestions for improvement were provided. These suggestions were reviewed by the other authors, and the FHIR StructureMaps were updated accordingly based on their agreement.

Lastly, the finalized FHIR StructureMaps were analyzed for their coverage of the SNOMED CT attributes listed above. Table 5 illustrates the number of attributes per category that could be successfully mapped in the respective profile (mappable). Depending on the category, up to 4 attributes could not be mapped to the native profiles (unmappable). For some of these attributes, existing FHIR extensions, such as those from HL7, can be introduced into the profiles to achieve a more complete mapping (with extension). Overall, between 56% (5/9) and 92% (12/13) of attributes (76.1% on average) can be mapped without modifications, depending on the category and profile. By introducing these extensions, coverage could be increased to an average of 93.5% (ranging from 67% [6/9] to 100% [eg, 9/9, 6/6, 13/13, for individual combinations]).

**Table 5:** The number of SNOMED CT elements that can be mapped directly to the profiles are represented by “mappable,” whereas elements that cannot be mapped are shown as "ummappable". FHIR^a^ offers extensions to map items that are not mappable by default, which could reduce the number of unmappable elements. The number of unmappable elements to represent by extension is shown in the last column.

Category: number of elements (total)	Profile type	Number of mappable elements (total)	Number of unmappable elements		
			Total	Could be mapped using extensions	
**Allergies: 9 elements**					
	NASHIP^b^	5	4	1	
	MII^c^	5	3	2	
**Disease due to allergies: 9 elements**					
	NASHIP	7	2	2	
	MII	7	2	2	
**Allergic reaction: 9 elements**					
	NASHIP	7	2	2	
	MII	7	2	1	
**Clinical finding (general): 6 elements**					
	NASHIP	4	2	2	
	MII	4	2	2	
**Procedure: 13 elements**					
	NASHIP	12	1	1	
	MII	12	1	1	

^a^FHIR: Fast Healthcare Interoperability Resources.

^b^NASHIP: National Association of Statutory Health Insurance Physicians.

^c^MII: Medical Informatics Initiative.

### Overall Evaluation With Existing PCEs

After validating several steps individually, the entire process of PCEtoFHIR was evaluated. To achieve a realistic scenario, 35 existing PCEs from the publication “Automatic Full Conversion of Clinical Terms into SNOMED CT Concepts” by Kate et al [45] were used, which are available online [50]. This publication presents a method for converting clinical texts into SNOMED CT PCEs, with the 35 PCEs manually created from clinical terms for a small-scale evaluation (see the “4.3. Evaluation Methodology” section in [45]).

To use this data set as input for PCEtoFHIR, the 35 provided PCEs were manually reviewed and automatically checked for syntactic and semantic correctness, as described previously. As a result, 2 PCEs were excluded from further processing: 1 for violating cardinality restrictions of the Concept Model and 1 for being equivalent to another. The remaining 33 PCEs include 23 from the top-level hierarchy *Clinical finding* and 10 from the top-level hierarchy *Procedure.* The following SNOMED CT attributes are used:

- *Clinical finding: Associated morphology, Finding site, Causative agent, Clinical course, Finding method*, and *Pathological process*
- *Procedure: Method, Procedure site—Direct, Using device*, and *Using substance*

These 33 PCEs were imported into PCEtoFHIR in bulk, bypassing the web front end but using the developed algorithm as usual. Each PCE was decomposed into a Superconcept and Delta, with FHIRPaths determined using the appropriate FHIR StructureMap. Based on these FHIRPaths, the corresponding attribute values were populated into FHIR resources, which had been prefilled with additional required data elements (such as references to a FHIR resource “Patient”). The resulting FHIR resources for each PCE were then stored on the local HAPI FHIR server [39].

**Figure 8:**
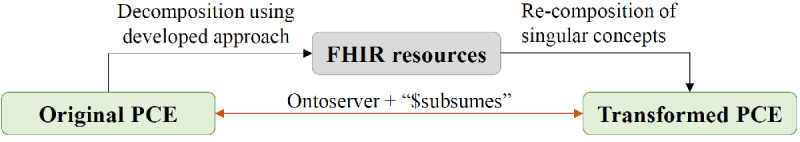
Validation process: The original PCE is decomposed by PCEtoFHIR as usual, and accordingly stored in FHIR resources. Based on this representation, the singular concepts are recomposed into a second PCE. The original and the recomposed PCE are compared. FHIR: Fast Healthcare Interoperability Resources; PCE: postcoordinated expression.

To determine whether all information contained within the original PCE has been successfully translated into the FHIR resources, the process shall now be reversed as shown in Figure 8. Based on the separate precoordinated SNOMED CT concepts spread across multiple FHIR elements, a PCE will be recomposed and compared with the original. For this, the values of the stored FHIR resources were extracted, and the respective StructureMap was applied in the reverse direction. The recomposed PCE uses the Superconcept as the focus concept, while its refinement consists of attribute relationships with the extracted SNOMED CT concepts as attribute values. For each of the extracted concepts, the corresponding SNOMED CT attribute must be determined according to the StructureMap’s mapping rules. As most mappings are inherently unidirectional [3], using them in the reverse direction can lead to several possible SNOMED CT attributes for some FHIRPaths. In these cases, the 3 rules listed in Textbox 2 are applied successively until a clear distinction is achieved.

**Rules applied for case distinction.**

- **By attribute value range**

For each of the attributes in question, a value range is defined by the SNOMED Clinical Terms Concept Model. This allows checking if the extracted concept falls within the respective value range and is thus a valid value for the corresponding attribute. If only a single value range matches, the correct attribute is identified.

- **By attribute hierarchy**

Some SNOMED CT attributes are organized hierarchically (eg, *Procedure device—Using device—Using access devices*). If multiple attributes from the same hierarchy are available, the most general attribute within this hierarchy is selected (in this case: *Procedure device*).

- **By occurrence heuristic**

The concept definitions of precoordinated concepts are analyzed to identify which SNOMED CT attribute is most frequently used for the given concept. The attribute with the highest statistical occurrence is selected.

Finally, each recomposed PCE was compared with the corresponding original PCE as a reference by testing their subsumption relationship via Ontoserver and the FHIR service $subsumes. For 32 of the 33 comparisons, the PCEs were evaluated as equivalent, indicating that no information was lost during the process. In the remaining comparison, the recomposed PCE was found to be a subclass of the original. Further analysis revealed that while some semantic precision was lost due to the required case distinctions, the FHIR representation achieved through PCEtoFHIR remained semantically equivalent to the original PCE. The approach thus proved successful, demonstrating the preservation of a PCE’s content.

## Discussion

### Principal Findings

This work aimed to develop an algorithm for generating alternative representations of SNOMED CT PCEs. The “Introduction” section discussed various reasons for the reluctance to use PCEs, including the preference for editing and saving individual codes. Precoordinated concepts offer the advantage of a single code representing a medical circumstance, along with a human-readable description. These aspects are seen as advantages when using precoordinated concepts but are not present in postcoordination. However, by identifying a Superconcept using OWL and semantic similarity, a precoordinated SNOMED CT concept that most accurately describes the PCE can be determined. The PCE, as a subconcept of this Superconcept, can then be effectively displayed and analyzed. In addition, the decomposition of the PCE results in individual codes that users are familiar with from practical experience. FHIR, recognized as a central interoperable data standard in health care, is gaining increasing importance both in Germany [51] and globally [51-53].

The calculation of semantic similarity is crucial for identifying the concept most similar to a PCE. In this work, the measure by Sánchez and Batet [34] was selected due to its suitability for SNOMED CT’s polyhierarchies and multiple inheritances. While this measure has been validated as effective, other approaches to semantic similarity calculation could also be considered. Although choosing an appropriate Superconcept is important, any remaining information is represented through the Delta, so alternative semantic similarity measures would have minimal impact on the overall functionality of PCEtoFHIR. However, PCEtoFHIR could be enhanced by allowing users to manually select or adjust the Superconcept.

As described above, the Superconcept and the Delta are stored in the relevant FHIR profiles, within their corresponding elements. For this purpose, the profiles of the NASHIP [4] and the profiles of the core data set of the MII [5] were utilized. However, the approach can also be adapted to other profiles. As shown in Table 5, depending on the content category and profile type, between 56% (5/9) and 92% (12/13) of attributes could be mapped directly to the respective profile without modifications, demonstrating a high coverage of content but also highlighting some gaps.

An analysis of the unmappable attributes revealed that these relations mostly concern very specific details that occur infrequently in precoordinated concept definitions. As PCEs are generally constructed based on existing concept definitions, they are unlikely to regularly utilize these highly specific attributes. SNOMED CT, however, provides the capability to include medical facts at such a granular level. By contrast, HL7 FHIR was designed with a pragmatic approach, focusing on the most prevalent information. This fundamental design discrepancy accounts for the majority of mapping challenges.

Nevertheless, as intended by the FHIR standard, suitable extensions from HL7 are available to appropriately represent some of these attribute relations (see Tables 4 and 5) and could be integrated into the profiles to extend their coverage of PCE content. Currently, the core data set lacks a profile for the FHIR resource AllergyIntolerance, which limits the representability of this content category. However, because the core data set is an ongoing modeling initiative, such a profile may be added in the future.

Apart from the unmappable attributes, the proposed mapping rules cannot always ensure an exact translation. In some cases, there is no precisely matching FHIR element for a specific attribute, or multiple attributes must be mapped to the same element (eg, *Using access device* and *Using device* can both only be recorded via the FHIR element *Device.type,* which is then referenced via *Procedure.usedReference*). As a result, some semantic precision may be lost. Therefore, it is advisable to store the original PCE in the metadata of the FHIR representation to ensure the preservation of the original meaning.

Nevertheless, the outlined difficulties in achieving a semantically equivalent representation through FHIR elements highlight the precision attainable through SNOMED CT’s postcoordination, underscoring its importance for detailed medical data description.

Several validations were conducted to ensure the correctness of both the individual processing steps and the overall functionality of PCEtoFHIR. Although a large data set from SNOMED International’s releases could be used to firmly validate the OWL expression generation, other evaluations required preexisting real-world PCEs of specific content categories, which are not readily available. The employed set of 35 PCEs meets these criteria and effectively facilitates our validation approaches. However, the limited number and semantic variance of these exemplary PCEs suggest that incorporating additional reference data could enhance the significance of the results.

Another validation included the manual review of the FHIR StructureMaps by a FHIR expert. This review revealed only minor inaccuracies, which were corrected in the current version of StructureMaps. Using these StructureMaps, the mapping for the considered categories was successfully completed and can be extended to other subhierarchies of SNOMED CT without difficulty. Hence, further possibilities of application may be considered, such as addressing the TermInfo problem. As mentioned, the choice between representing medical facts in a terminology or an information model often varies and depends on the intended use. For example, “Fracture of the left femur” can either be represented using a single PCE as the FHIR element *Condition.code* like

- *Condition.code: 71620000:{363698007=722738000}*
*(Fracture of femur : {Finding site = Structure of bone of left femur})*

or by splitting the semantic meaning up into two precoordinated SNOMED CT concepts using further element-code-combinations of the FHIR resource *Condition*:

- *Condition.code: 71620000 |Fracture of femur (disorder)|*
- *Condition.bodySite: 722738000 |Bone structure of left femur (body structure)|.*

This variability in expressing medical facts was leveraged in the presented approach, enabling flexible transformation between terminology-focused and information model–focused representations. This allows for an alternative when replacing PCEs and may help address some challenges related to the TermInfo problem. By enabling flexible switching between different expression methods, semantic interoperability is maintained regardless of the representation paradigm used in an electronic health record. Additionally, this approach facilitates the plausibility check of recorded medical information by allowing the integration of disjointed elements (eg, scattered across various FHIR elements) into a single *interpretable* expression.

Despite the general ambiguity regarding the scope of semantic versus structural standards, some specific recommendations do exist, such as the suggestion that “contextual meaning should rather be represented via the information model” [54]. Contrarily, concepts within the SNOMED CT hierarchy *Situation with explicit context* encompass contextual information that extends beyond the typical scope of a terminology, such as suspected diagnoses, procedures not done, or family history facts. This additional contextual information can affect logical conclusions. An explanation of the reasons behind this, involving epistemological versus ontological components of meaning, is beyond the scope of this paper (see [3,13]). Based on the logical definitions of concepts directly related to PCEs, the approach presented in this paper enables the extraction of problematic pieces of information and their storage within separate elements of the information model. As a result, a concept like *165008002 |Allergy testing not done (situation)|* could be represented by separating the epistemological aspect “not done” into the suitable FHIR element *Procedure.status*:

- *Procedure.code: 252512005 |Allergy test (procedure)|*
- *Procedure.status: not-done* (according to the required HL7 FHIR ValueSet).

In this way, the integrity of SNOMED CT’s hierarchies may be preserved.

### Conclusions

The use of PCEs greatly enhances SNOMED CT’s capacity to capture medical details comprehensively. However, despite its advantages, postcoordination has not yet been widely adopted in routine data collection. To address this, PCEtoFHIR offers a solution to ensure semantic interoperability between systems that are adept at postcoordination and those that are not, by leveraging the globally accepted HL7 FHIR standard to provide an alternative representation. State-of-the-art techniques in description logic and terminology services are integrated into a largely automated web application that decomposes PCEs into their core components. Validation of both individual steps and the overall process confirms the approach’s functionality. PCEtoFHIR is designed and implemented modularly, positioning it for future adaptations in the evolving landscape of health informatics. In addition to straightforward extensions to other SNOMED CT hierarchies or FHIR profiles by adding more StructureMaps, the algorithm can be adapted to work with other information models, such as openEHR or relational databases. The approach also holds the potential for addressing further challenges in semantic and structural standards, such as the TermInfo problem. By reversing the processing direction—from FHIR elements back to PCE—meaningful SNOMED CT-based analyses could be facilitated.

## Abbreviations

CSIRO: Commonwealth Scientific and Industrial Research Organization
DAG: directed acyclic graph
ECL: Expression Constraint Language
FHIR: Fast Healthcare Interoperability Resources
HL7: Health Level 7
KBV: Kassenärztliche Bundesvereinigung
LOINC: Logical Observation Identifiers Names and Codes
MII: Medical Informatics Initiative
NASHIP: National Association of Statutory Health Insurance Physicians
OWL: Web Ontology Language
PCE: postcoordinated expression
RIM: Reference Information Model
SNOMED CT: SNOMED Clinical Terms
